# The impact of assumptions regarding vaccine-induced immunity on the public health and cost-effectiveness of hepatitis A vaccination: Is one dose sufficient?

**DOI:** 10.1080/21645515.2016.1203495

**Published:** 2016-07-18

**Authors:** Desmond Curran, Marc de Ridder, Thierry Van Effelterre

**Affiliations:** a Economics Department, GSK Vaccines, Wavre, Belgium; b GSK Vaccines, Wavre, Belgium

**Keywords:** cost-effectiveness, hepatitis A, Immune memory, mathematical model, number of doses, vaccination

## Abstract

Hepatitis A vaccination stimulates memory cells to produce an anamnestic response. In this study, we used a mathematical model to examine how long-term immune memory might convey additional protection against clinical/icteric infections. Dynamic and decision models were used to estimate the expected number of cases, and the costs and quality-adjusted life-years (QALYs), respectively. Several scenarios were explored by assuming: (1) varying duration of vaccine-induced immune memory, (2) and/or varying levels of vaccine-induced immune memory protection (IMP), (3) and/or varying levels of infectiousness in vaccinated individuals with IMP. The base case analysis assumed a time horizon of 25 y (2012 – 2036), with additional analyses over 50 and 75 y. The analyses were conducted in the Mexican public health system perspective. In the base case that assumed no vaccine-induced IMP, the 2-dose hepatitis A vaccination strategy was cost-effective compared with the 1-dose strategy over the 3 time horizons. However, it was not cost-effective if we assumed additional IMP durations of at least 10 y in the 25-y horizon. In the 50- and 75-y horizons, the 2-dose strategy was always cost-effective, except when 100% reduction in the probability of icteric Infections, 75% reduction in infectiousness, and mean durations of IMP of at least 50 y were assumed. This analysis indicates that routine vaccination of toddlers against hepatitis A virus would be cost-effective in Mexico using a single-dose vaccination strategy. However, the cost-effectiveness of a second dose depends on the assumptions of additional protection by IMP and the time horizon over which the analysis is performed.

## Introduction

Hepatitis A Virus (HAV) affects more than 126 million people worldwide annually according to the World Health Organization (WHO).^1^ It is transmitted mainly by the fecal-oral route, either by direct contact with an infectious person or by ingestion of contaminated food or water.^1^ The probability of developing a hepatitis A icteric infection (i.e., jaundice) increases with age (see supplemental text for further detail). Furthermore, the probability of hospitalization and death, given an icteric infection, increases with age.^2^ As such, an acute hepatitis A viral infection is usually asymptomatic in young children and remains undiagnosed due to lack of jaundice. Older children and adults with acute infections, however, may have a mild infection or, may develop serious complications.

In regions of high endemicity, nearly all children become infected early in life, and therefore most cases are asymptomatic. Consequently, vaccination is not recommended by the WHO in countries with high endemicity.^1^ In regions of moderate endemicity, the mean age of HAV infection increases leading to an increase in the number of symptomatic cases, i.e., as the clinical severity of hepatitis A increases with age. Therefore, the WHO recommends that vaccination against HAV should be integrated into the national immunization schedule for children aged ≥1 y if there is a change in the endemicity of hepatitis A from high to intermediate, and after consideration of cost-effectiveness. The WHO concluded that national immunization programs may consider inclusion of single-dose inactivated hepatitis A vaccines in immunization schedules.^1^ Nevertheless the WHO recommended that until further experience has been obtained with a single-dose schedule, a 2-dose schedule is preferred, in for example, immunocompromised individuals.

Vaccine-induced immunity is typically evaluated by means of the antibody titer.^3^ However, The International Consensus Group on Hepatitis A Virus Immunity concluded that the underlying immune memory provides protection far beyond the duration of anti-HAV antibodies.^4^ Several studies have demonstrated that exposure to a challenge dose of hepatitis A stimulates memory cells to produce an anamnestic response.^5,6,7^ Ott and Wiersma performed a literature review which included a summary of the available evidence on long term seropositivity rates (SPR) for a single dose of hepatitis A.^8^ There were limited data on long-term protection (i.e., beyond 6 y) with a single dose of hepatitis A. One study included 26 adult travelers who received a booster dose 98–128 months after the primary vaccination.^9^ 53.8% of subjects had protective levels of anti-HAV antibodies (≥10 mIU/ml) pre-booster compared with 100% post-booster dose, supporting the hypotheses that the initial vaccination introduced immunological memory. While antibodies are proficient at preventing infection, cell-mediated immunity typically responds to pathogens once they have infected cells, leading to improved disease control and consequently less severe diseases. These studies indicating a single hepatitis A dose is sufficient to establish a long-lasting immune memory response lead to questions concerning the need for further vaccine doses.

In 2005, the Argentinian National Ministry of Health, in agreement with national experts, introduced a single dose of inactivated hepatitis A vaccine at 12 months of age.^10^ Several studies have reported the success of the 1-dose program, such as the decline in HAV-associated fulminant hepatic failures and liver transplants in children.^11^ However there are limited data on long-term protection (i.e., beyond 6 y) with a single dose of hepatitis A.^12^ In contrast, longitudinal observed data of antibody levels over a period of 17 y, supplemented by a kinetic model of antibody decline suggest that vaccine-induced immunity will persist for decades after a 2-dose series.^13,14,15^

To date cost-effectiveness models of vaccination against hepatitis A have focused on disease prevention only. In this study we examined how vaccine-induced long-term immune memory might convey additional protection against clinical/icteric infections using a mathematical model. We explored various scenarios by assuming: (1) varying duration of vaccine-induced immune memory, (2) and/or varying levels of immune protection, (3) and/or varying levels of infectiousness in vaccinated individuals with IMP. Mexico was chosen as the country for this analysis as recent seroprevalence studies indicate that the seroprevalence pattern for HAV has shifted from high to intermediate endemicity levels.^16^

This analysis may assist policy makers to evaluate the public health impact and cost-effectiveness of 1-dose versus 2-dose of hepatitis A vaccination in terms of prioritization in a limited resource environment.

## Results

**Figure 1.**
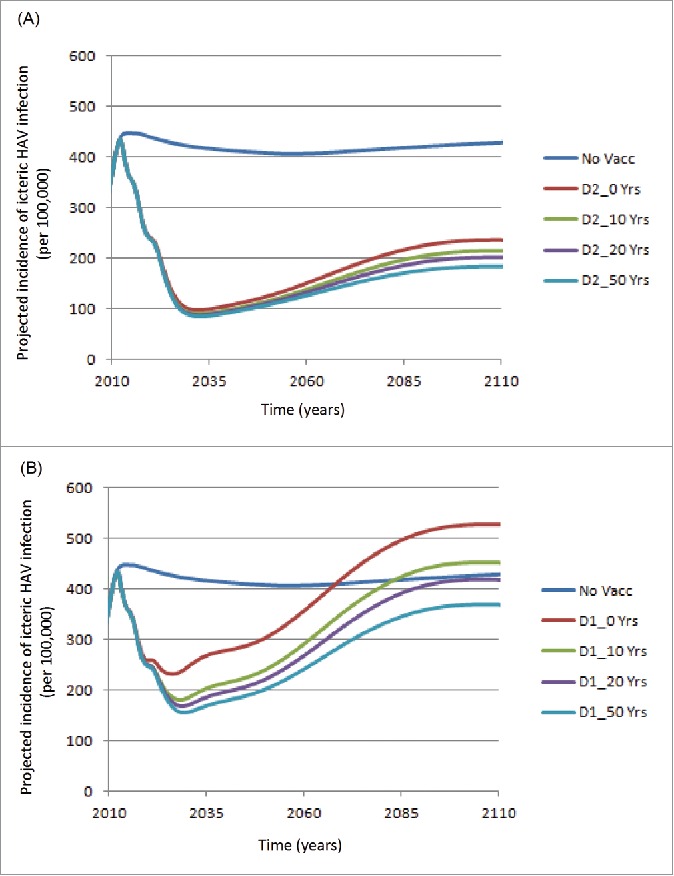
Projected icteric HAV incidence (per 100,000) over time: Assuming 50% reduction in the probability of icteric Infections / 50% reduction in infectiousness for (A) 2-dose, and (B) 1-dose vaccination schedule (No Vacc: No vaccination; D1: Dose 1; D2: Dose 2; Yrs: Years of Immune Memory Protection).

Figure 1A shows the projected incidence of icteric HAV infections over time, assuming a 50% reduction in the risk of icteric infections and a 50% reduction in infectiousness using a 2-dose schedule and different mean durations of IMP. A rapid decline in the incidence of icteric infections over the first 20 y after vaccine introduction was projected, with a subsequent increase over the next 60 y. During this period the assumptions surrounding IMP have limited impact. Similarly, Fig. 1B presents the results for a 1-dose vaccination schedule. It is noteworthy that, during the first 10 y after vaccine introduction, the reduction in incidence is similar to the outcome of the 2-dose schedule. Thereafter, the incidence is highly dependent on the assumptions regarding IMP. If no additional IMP is assumed then the projected incidence increases to values higher than a no vaccination strategy after approximately 60 y. Similarly, if a mean duration of IMP of 10 y is assumed, the projected incidence increases to values higher than a no vaccination strategy after approximately 70 y. The best results (i.e., with a mean duration of IMP of 50 y), in terms of reduced incidence, observed with a 1-dose strategy, are not as good as the worst scenario with a 2-dose strategy (i.e., with a mean duration of IMP of 0 y).

The results in Fig. 1 should be put into context. For example, not much variation is seen over time in the HAV incidence in the no vaccination strategy. However, much is happening over the 100-y time period, i.e., changing demographics, and an age shift in the mean age of icteric infection due to decreased transmission. Table 1 presents mean age of icteric infection for the 3 vaccination strategies assuming no additional IMP protection. In the no vaccination strategy the mean age of icteric infection shifted from 11.4 y in 2012 to 20.8 in 2086. The age shifts are greater in the 2-dose strategy compared to the 1-dose strategy.

**Table 1.** Mean age of icteric infection with 3 different vaccination strategies assuming no protection against icteric infection after vaccine protection has waned (no IMP).

Year	Age	No Vaccination	1-Dose	2-Dose
2012	Mean	11.4	11.4	11.4
	Median	10	10	10
	%>=60	0.26%	0.26%	0.26%
2036	Mean	16.4	20.0	26.6
	Median	11	15	19
	%>=60	1.67%	2.04%	4.22%
2061	Mean	20.6	29.5	36.6
	Median	12	19	40
	%>=60	6.17%	7.42%	14.13%
2086	Mean	20.8	37.9	43.8
	Median	12	41	47
	%>=60	7.43%	20.90%	28.31%

IMP, Immune Memory Protection.

Table 2 presents the outcomes averted for 1-dose and 2-dose vaccine strategies compared with no vaccination using the same assumptions as in Fig. 1. The 1-dose strategy over a 25 y time horizon, assuming IMP durations of 10 to 50 y, progressively resulted in more icteric cases averted, lower costs (i.e., cost savings) and greater QALY gains. The 50-y time horizon offered similar results. However, a negative impact of the age shift was observed for a longer time horizon (i.e., 75 y). For example, assuming no IMP, the 1-dose strategy resulted in a reduction of approximately 8.3 million icteric cases and 560,350 reported icteric cases, but led to more hospitalizations and greater number of deaths compared with a no vaccination strategy. Assumptions of longer IMP durations improved the results (see Table 2, the increased number of hospitalizations averted) but even with a mean duration of 50 y IMP, a greater number of deaths were projected.

**Table 2.** Outcomes comparing “1-dose” and “2-dose” vs. “no vaccination,” assuming immune memory protection (IMP) with a 50% reduction in the probability of icteric Infections / 50% reduction in infectiousness in vaccinated subjects who subsequently develop hepatitis A.

		Avoided				Discounted		ICUR^*^
Time Horizon	Duration of IMP (Years)	Icteric	Reported Icteric	Hospital	Deaths	Medical Costs 5%^*^	QALYs 5%^*^	5%
1-Dose								
25	0	5,445,631	367,012	16,123	1,063	386,807,257	175,968	2,198
	10	6,633,884	447,095	20,999	1,274	−210,864,060	187,266	−1,126
	20	6,925,717	466,763	22,289	1,331	−356,123,027	190,103	−1,873
	50	7,160,804	482,607	23,354	1,379	−473,129,496	192,421	−2,459
50	0	9,440,094	636,221	19,005	675	529,979,533	226,256	2,342
	10	13,099,558	882,853	35,496	1,726	−579,868,059	246,835	−2,349
	20	14,184,798	955,994	41,080	2,168	−893,174,768	252,897	−3,532
	50	15,165,974	1,022,121	46,423	2,643	−1,168,437,562	258,329	−4,523
75	0	8,314,334	560,350	−22,322	−11,493	1,035,887,612	228,499	4,533
	10	14,634,695	986,315	8,479	−8,750	−246,186,699	252,239	−976
	20	16,898,313	1,138,873	21,996	−6,893	−643,957,219	260,027	−2,477
	50	19,330,441	1,302,788	37,981	−4,229	−1,026,948,109	267,759	−3,835
2-Dose								
25	0	8,407,350	566,619	28,872	1,630	3,133,939,181	211,341	14,829
	10	8,565,222	577,259	29,564	1,661	3,048,793,184	212,972	14,315
	20	8,607,590	580,114	29,761	1,670	3,026,561,059	213,395	14,183
	50	8,641,854	582,424	29,923	1,677	3,008,671,327	213,735	14,077
50	0	19,562,853	1,318,451	69,223	4,318	2,840,214,945	296,798	9,570
	10	20,089,705	1,353,959	72,040	4,545	2,677,415,604	299,883	8,928
	20	20,268,413	1,366,003	73,091	4,646	2,626,801,053	300,844	8,731
	50	20,433,553	1,377,133	74,101	4,754	2,582,017,836	301,699	8,558
75	0	28,162,411	1,898,024	90,034	2,242	2,956,474,568	316,268	9,348
	10	29,319,398	1,976,000	97,241	3,268	2,748,501,992	320,274	8,582
	20	29,829,969	2,010,411	100,834	3,913	2,673,085,743	321,762	8,308
	50	30,403,307	2,049,051	105,095	4,767	2,597,436,999	323,286	8,034

* Mexican threshold: 132,465 Mexican Pesos/QALY.

ICUR: Incremental cost-utility ratio.

QALYs: quality-adjusted life-years.

IMP: Immune Memory Protection.

A 2-dose strategy leads to more consistent results between scenarios (see Fig. 1 and Table 2). For example, the total number of deaths averted ranged from 1,630 to 4,767. While this strategy was never shown to be cost-saving it was always cost-effective compared with a no vaccination strategy (ICUR range: 8,034 to 14,829 MXN/QALY).

Table 3 presents the ICURs comparing a 2-dose vs. a 1-dose strategy. In the base case, assuming no IMP, the “2-dose” strategy is cost-effective (i.e., less than the Mexican threshold of 132,465 MXN/QALY) compared with a 1-dose strategy over the 3 time horizons.^17^ However, focusing on the 25-y time horizon, it is no longer cost-effective if we assume additional IMP durations of at least 20 y, assuming a 50% reduction in the probability of icteric infections / 50% reduction in infectiousness. Exploring longer time horizons, i.e., 50 and 75 y, the 2-dose strategy is always cost-effective compared with the 1-dose strategy.

**Table 3.** ICUR comparing “2-dose” versus “1-dose” of Hepatitis A vaccination.

		Time Horizon		
Year	Duration of IMP (Years)	25 Y	50 Y	75 Y
50% reduction in the probability of icteric infections / 50% reduction in infectiousness	0102050	77,662126,800145,229163,357	32,75061,40373,41486,476	21,88244,01753,73065,272
100% reduction in the probability of icteric infections / 75% reduction in infectiousness	0102050	77,662176,637225,542280,651	32,75090,964123,751166,959	21,88266,17591,866130,082

* Mexican threshold: 132,465 Mexican Pesos/QALY.

ICUR: Incremental cost-utility ratio.

IMP: Immune Memory Protection.

**Figure 2.**
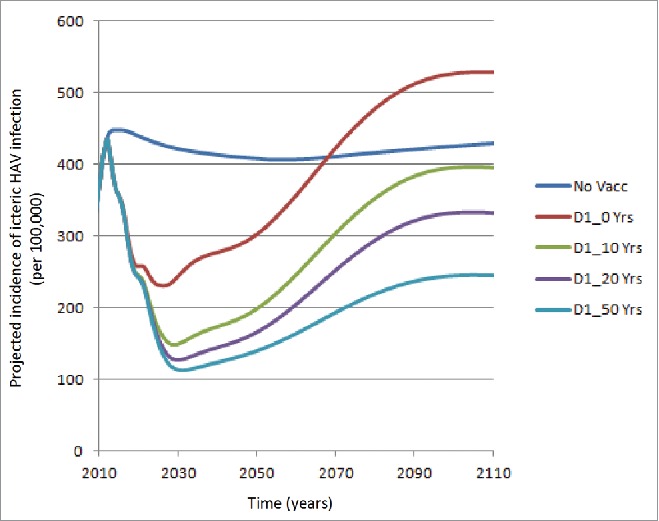
Projected icteric HAV incidence (per 100,000) over time: Assuming 100% reduction in the probability of icteric infections / 75% reduction in infectiousness (No Vacc: No vaccination; D1: Dose 1; D2: Dose 2; Yrs: Years of Immune Memory Protection).

Fig. 2 explores a more optimistic scenario using a 1-dose schedule as compared with Fig. 1B, i.e., assuming 100% reduction in the probability of icteric infections / 75% reduction in infectiousness. It may be seen that incidence rates are lower for the various curves, i.e., assuming a mean IMP duration from 10 to 50 y. Under the scenario of a mean IMP duration of 50 y, the curve is quite similar to that presented for a 2-dose schedule assuming no IMP.

Table 4 presents the outcomes averted for the 1-dose and 2-dose vaccine strategies compared with no vaccination using the same assumptions as in Fig. 2. Although the results demonstrated an improvement from the Table 2 results, particularly for the 1-dose strategy, i.e., there was always a reduction in hospitalizations, assuming an IMP of at least 10 y, a negative impact on deaths was observed over a 75-y time horizon over shorter durations of IMP, e.g., 10 and 20 y.

**Table 4.** Outcomes comparing “1-dose” and “2-dose” vs. “no vaccination,” assuming immune memory protection (IMP) with a 100% reduction in the probability of icteric infections / 75% reduction in infectiousness in vaccinated subjects who subsequently develop hepatitis A.

		Avoided				Discounted		ICUR
Time Horizon	Duration of IMP (Years)	Icteric	Reported Icteric	Hospital	Deaths	Medical Costs 5%^*^	QALYs 5%^*^	5%
1-Dose								
25	0	5,445,631	367,012	16,123	1,063	386,807,257	175,968	2,198
	10	7,361,578	496,138	24,098	1,396	−571,047,344	193,672	−2,949
	20	7,784,179	524,620	26,021	1,482	−784,049,006	197,888	−3,962
	50	8,100,662	545,949	27,501	1,549	−945,173,386	201,169	−4,698
50	0	9,440,094	636,221	19,005	675	529,979,533	226,256	2,342
	10	15,524,008	1,046,251	47,292	2,445	−1,288,450,226	259,225	−4,970
	20	17,210,554	1,159,917	56,455	3,198	−1,771,304,714	268,707	−6,592
	50	18,669,343	1,258233	64,902	3,998	−2,176,543,044	276,937	−7,859
75	0	8,314,334	560,350	−22,322	−11,493	1,035,887,612	228,499	4,533
	10	19,071,476	1,285,335	32,181	−6,531	−1,086,663,916	266,979	−4,070
	20	22,798,487	1,536,519	55,671	−3,139	−1,717,998,455	279,521	−6,146
	50	26,779,394	1,804,815	83,320	1,762	−2,313,552,322	291,869	−7,927
2-Dose								
25	0	8,407,350	566,619	28,872	1,630	3,133,939,181	211,341	14,829
	10	8,663,801	583,903	29,990	1,678	2,997,013,179	213,871	14,013
	20	8,729,110	588,304	30,292	1,691	2,962,884,378	214,501	13,813
	50	8,780,781	591,787	30,535	1,701	2,935,952,860	214,998	13,656
50	0	19,562,853	1,318,451	69,223	4,318	2,840,214,945	296,798	9,570
	10	20,444,563	1,377,875	73,996	4,711	2,573,792,172	301,684	8,531
	20	20,735,444	1,397,479	75,742	4,887	2,493,520,866	303,170	8,225
	50	21,003,259	1,415,529	77,418	5,076	2,422,999,049	304,486	7,958
75	0	28,162,411	1,898,024	90,034	2,242	2,956,474,568	316,268	9,348
	10	30,184,910	2,034,332	102,915	4,164	2,608,027,247	322,811	8,079
	20	31,073,553	2,094,223	109,326	5,371	2,482,846,206	325,249	7,634
	50	32,075,102	2,161,723	116,949	6,967	2,356,507,179	327,771	7,189

* Mexican Threshold: 132,465 Mexican Pesos/QALY.

ICUR: Incremental cost-utility ratio.

QALYs: quality-adjusted life-years.

IMP: Immune Memory Protection.

The 2-dose strategy again led to more consistent results. For example, the total number of deaths avoided ranges from 1,630 to 6,967 (ICUR range: 7,189 to 14,829 MXN/QALY). Comparing the ICURs for the 2-dose versus the 1-dose strategy (see Table 3), the 2-dose strategy was not cost-effective, assuming an IMP of at least 10 y over a 25-y time horizon or an IMP of at least 50 y over a 50-y time horizon. However, the 2-dose strategy was always cost-effective over a 75 y time horizon.

## Discussion

A systematic review suggested that universal hepatitis A vaccination of infants, children and adolescents in middle-income intermediate endemicity countries (e.g., Argentina, Brazil, Chile, China and Egypt) is cost effective.^18^ In this paper we explored the potential impact of HAV vaccine immune memory on the modeled reduction of hepatitis A disease in vaccinated subjects.

After vaccination, as circulating serum antibodies decline over time, exposure to the HAV stimulates memory T-cells to produce an anamnestic response (similar to that observed in challenge dose studies).^13^ Memory T-cells are a subset of antigen-specific T-cells that persist for a long time after hepatitis A vaccination.^19^ Upon re-exposure to hepatitis A, the induced memory T-cells quickly expand to orchestrate an anamnestic humoral response.^19,20^ This immune response may not be able prevent a hepatitis A infection but most likely infectiousness and the probability of an icteric hepatitis A infection are reduced. Furthermore, in a recent study, the authors concluded that a single dose of inactivated hepatitis A vaccine promotes HAV-specific memory cellular response similar to that induced by a natural infection.^21^

Traditionally, SPR rates have been assessed using cut-off levels of ≥10 mIU/ml and ≥20 mIU/ml. Hatz et al. presented SPR rates at 98–128 months, after receiving the primary dose, of 80.8%, 53.8% and 50.0% assuming cut off values of ≥6, ≥10 and ≥20 mIU/ml, respectively. Studies of passively transferred antibodies have shown that even antibody concentrations just above the detection cut-off are associated with an absence of clinical infection.^22,23^ The SPR values presented by Hatz et al. using a ≥ 6 mIU/ml cut-off value are similar to the efficacy estimates, for a single-dose, at 10 y included in our model (i.e., 82.6%). Van Effelterre et al. previously investigated the impact of assumptions of waning of vaccine efficacy on epidemiological outcomes in Mexico. The subsequent paper of Carlos et al. 2015 presented a scenario analysis with various assumptions regarding waning of single-dose efficacy after 10 y (i.e., 0.62%, 1%, 1.5%, 1.75%, 2.67%) from a cost-effectiveness perspective.^19^ The scenario analysis suggested that when the waning rate was ≤1% from year 11 onwards, the single dose lead to cost savings when compared with no vaccination. Assuming a lower waning rate of 0.62% resulted in a 2-dose schedule not being cost-effective when compared with a single dose. Higher waning rates for single-dose, from year 11 onwards, always lead to the 2-dose schedule being cost-effective when compared with a single-dose. In this analysis we use the base case assumption of 2.67% waning after 10 y with a single-dose.

The results for a 1-dose vaccine strategy appear promising especially over a short time horizon (e.g., 25 y) even without additional IMP assumptions. However, exploring a longer time horizon (i.e., 75 y), with a 1-dose strategy, may result in more deaths than a no vaccination strategy. This is primarily observed because of the age shift in the mean age of icteric infections and an associated increase in disease severity in older adults. However, if one assumes that IMP duration lasts, on average, for 50 y or more, then a 1-dose strategy assuming a 100% reduction in the probability of icteric infections and a 75% reduction in infectiousness in vaccinated subjects would lead to an overall reduction in the number of hepatitis A deaths and cost savings.

Many economic analyses of at-birth vaccination strategies are carried out over a lifetime time horizon. Based on the shorter time horizon, a 1-dose vaccination strategy may appear to be the most attractive option. However, economic analyses should take into account both the short and long-term impacts of an intervention. The 2-dose vaccination strategy offers more certainty over a longer period of time, especially related to hospitalizations and deaths. In addition, there are substantial data demonstrating that 2-dose of hepatitis A vaccination offers protection against HAV lasting decades. A recent modeling study, based on 17–20 y actual data, predicted that 90% of subjects would remain seropositive 40 y after vaccination with a 2-dose schedule.^24^ However, much uncertainty surrounds the duration of protection offered by a 1-dose schedule, i.e., almost no data are available.

Argentina's Ministry of Health implemented universal hepatitis A vaccination with a single dose schedule at 12 months of age.^11^ As much uncertainty remains concerning the duration of protection with a 1-dose schedule, it is imperative that appropriate monitoring systems are in place.^12^ In addition to seroprevalence studies, it will be particularly important to monitor the impact on clinical events such as hospitalizations, fulminant disease, liver transplants and deaths related to hepatitis A. As was shown in Tables 2 and 4, although there may be large reductions in the overall number of reported icteric infections after vaccination with a 1-dose schedule, this could still potentially translate into increased numbers of hospitalizations and deaths compared with a no vaccination strategy. On the other hand, there may be a dramatic increase in the number of infections over time due to antibody decline after vaccination. However, if the IMP assumption is upheld, many of these infections may either be asymptomatic or anicteric or even icteric hepatitis A cases that remain unreported due to the mild nature of the infection. In either situation it will be important to ensure that proper surveillance systems are put in place to monitor not only the presence of infections but to assess the type and severity of infections.

As Argentina was the first country to introduce a 1-dose universal hepatitis A vaccination, many other countries (in particular in the Latin American region) are waiting to see the public health impact of this strategy over time. If this strategy continues to show promising results then other countries will most likely follow suit.

In this modeling exercise we chose to use the assumption that coverage for the 1-dose schedule was 80%. This may be conservative. For example, Cervio *et al.* reported coverage rates of 98% in Argentina in 2006 for 1 dose of hepatitis A vaccine.^11^ Consequently, the results observed in Argentina are more impressive than those presented here for 1-dose (see Fig. 1B). Cervio *et al.* reported that no cases of fulminant hepatitis failure cases related to hepatitis A were reported from November 2006–December 2008.^11^ We also assumed that the coverage for the second dose was 85% of the first dose (i.e., 68%).

The current analysis has a number of limitations. First, the initial dynamic model was calibrated on epidemiological data at a time prior to vaccination when most infections took place at younger ages (i.e., <20 y of age). The data are therefore not very informative about the contact pattern (e.g., who acquires infection from whom) for HAV transmission, especially in older individuals. In particular, the lower transmission in individuals above 20 y of age in the transmission model reflected that youngest individuals were probably the most influential source for HAV transmission in Mexico (both in terms of being infectious to HAV and being susceptible to be infected by HAV), given the main fecal-oral route of transmission. However, the high level of uncertainty about the role of adults in HAV transmission has to be acknowledged, as the magnitude of the contribution of adults to HAV transmission is not well known. Further data on the long-term impact of a single-dose of HAV is required.

## Methods

A previously published dynamic model of hepatitis A in Mexico was adapted to account for a hypothetical efficacy against icteric infection, called immune memory protection (IMP) in the sequel.^25^ The original model was adapted by adding a new IMP state wherein the vaccinated individuals were partially protected by IMP after the vaccine efficacy against infection had waned. In that IMP state, the vaccinated individuals were assumed to have the same risk of being infected with HAV as the non-vaccinated individuals. However, individuals in the IMP state, if infected, have a lower risk of icteric infection and are potentially less infectious, as compared with non-vaccinated individuals. The individuals were assumed to stay in the IMP state for a variable mean duration of time (10, 20 and 50 y). See supplemental text for more detail.

In the IMP state, the probability of an icteric infection was assumed to be reduced as compared with non-vaccinated individuals, with a varying reduction of 0% and 100% by 50% increment. Similarly, individuals were assumed to be less infectious in the IMP state as compared with non-vaccinated individuals, with a reduction of 0% to 75% by 25% increment.

The vaccine efficacy against HAV infection was assumed to be an all-or-none protection against HAV infection in 97% of vaccinated individuals after dose 1 and in 99% of vaccinated individuals after dose 2. For the 2-dose schedule, the model assumed an annual waning rate of 0.12% per year during the first 25 y, and 0.62% thereafter. For the 1-dose schedule, an annual waning rate of 1.62% during the first 10 y was assumed and 2.67% thereafter (see supplemental text for more detail).

The economic analysis was conducted from the Mexican public health system perspective and the projected costs and outcomes in the total Mexican population of all ages were considered. The time horizon of the study was 25 y (from 2012 to 2036), 50 y (from 2012 to 2061) and 75 y (from 2012 to 2086). Both costs and benefits were discounted at an annual rate of 5% after the first year. All costs are expressed in 2012 Mexican pesos (MXN).

For each of the 3 alternative interventions considered (no vaccination, 1-dose HAV vaccination or 2-dose HAV vaccination), the model projects the expected number of HAV cases and the number of icteric HAV cases in each year. An icteric case may either be reported or unreported to the health system. It was assumed that 95% of unreported icteric cases require medical care and use of medical resources. All reported cases are assumed to consume medical resources. Reported cases are classified according to the type of treatment (e.g., ambulatory care or hospitalization) and clinical course (e.g., some cases develop fulminant hepatitis and liver failure, which in turn may result in a liver transplant or death). Further details regarding the economic model and key model inputs are provided in the recent publication (see table 5 in Carlos *et al.*)^19^

The epidemiological model was developed in *Matlab* (Version 7.9.0) and the health economic model in *SAS* (Version 9.2).

## Supplementary Material

Supplementary files

## Abbreviations

HAV: Hepatitis A Virus
QALY: quality-adjusted life-year
IMP: Immune Memory Protection
ICUR: Incremental Cost-utility Ratio
WHO: World Health Organization
MXN: Mexican peso
