# Supplementary material for: The impact of assumptions regarding vaccine-induced immunity on the public health and cost-effectiveness of hepatitis A vaccination: Is one dose sufficient?
Source: Hum Vaccin Immunother. 2016 Jul 18;12(11):2765–71. doi: 10.1080/21645515.2016.1203495 (PMC5137541; doi:10.1080/21645515.2016.1203495)
Supplement: Supplementary files [file khvi-12-11-1203495-s001.docx]

***Probability of developing jaundice (i.e. icteric infection)***

Armstrong and Bell estimated the age-specific probability of developing jaundice during acute Hepatitis A Virus (HAV) infection to be P = 0.852 × (1-exp[0.01244**a*^1.903^]), where “a” is the age at the time of infection, in years. ^1^ Based on this formula, Supplementary Figure 1 presents the probability of icteric infection by age. As it may be seen, under a no-vaccination scenario, the probability of observing an icteric infection in infants is very low. However, the probability increases with age until approximately the age of 20 years after which the probability reaches a plateau. This observation explains a number of phenomena related to HAV infection. Firstly, as the socio-economic and sanitation level of a country improves, the endemicity of hepatitis A shifts from high, to intermediate to low. When this occurs, the average age of infection increases, thus increasing the probability of an infection being an icteric infection. As such the burden of disease is generally greater in intermediate endemicity areas compared with high endemicity areas.

**Supplementary Figure 1: Probability of Icteric Infection by Age based on the Model from Armstrong and Bell ^1^**


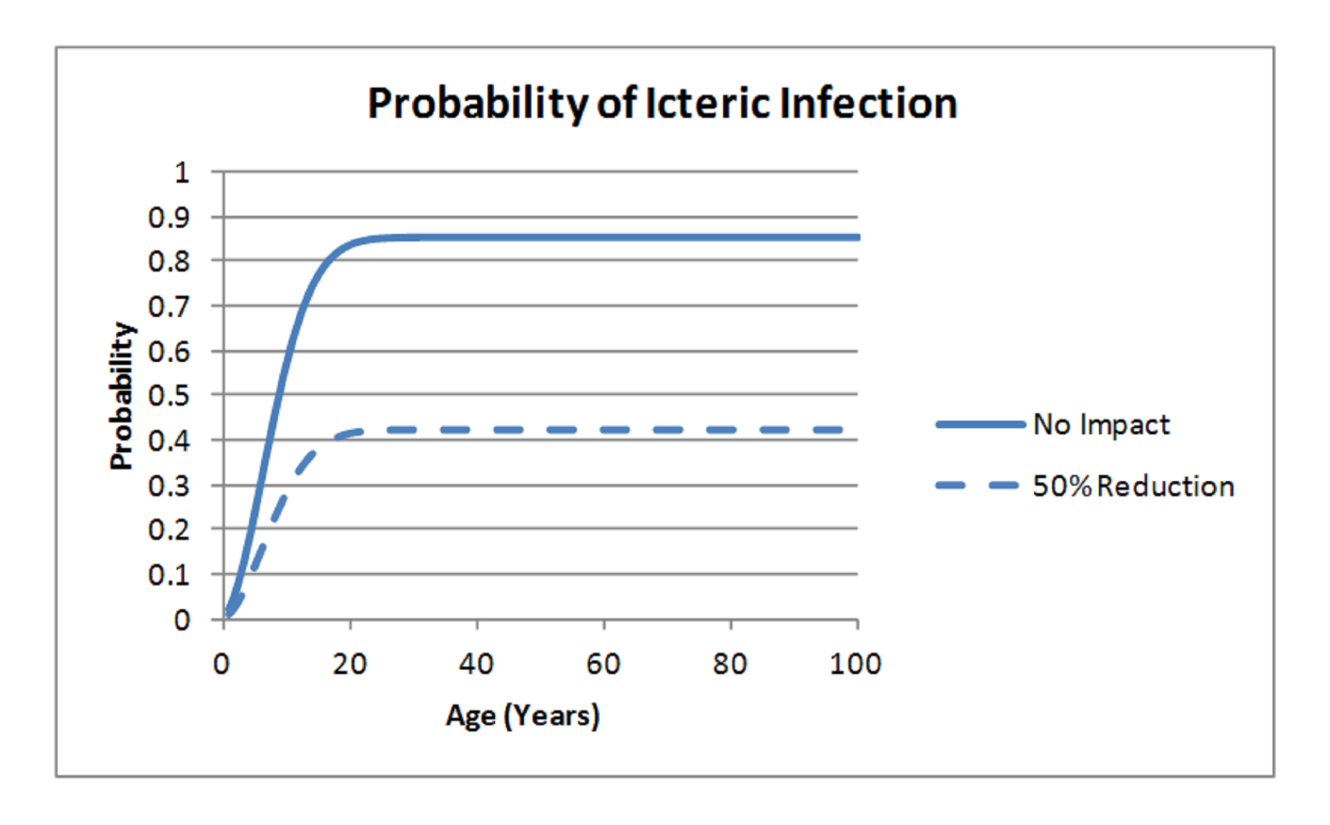


In the Immune Memory Protection (IMP) state, the risk of icteric/clinical infection was assumed to be reduced as compared to non-vaccinated individuals, with a reduction varying between 0% and 100% by 50% increment.

***Vaccine Efficacy***

The vaccine efficacy against HAV infection was assumed to be an all-or-none protection against HAV infection in 97% of vaccinated individuals after dose 1 and in 99% of vaccinated individuals after dose 2. For the 2-doses schedule, the model assumed an annual waning rate of 0.12% per year during the first 25 years (i.e. leading to 97% of individuals still protected at year 25 post-vaccination). Thereafter, an annual waning rate of 0.62% was used, based on a previous publication with inputs from an expert panel ^2^. In the absence of data on the duration of vaccine protection after a single dose, an annual waning rate of 1.62% during the first 10 years and 2.67% thereafter was assumed, consistent with a prior model of HAV ^2^ (Supplementary Figure 2).

**Supplementary Figure 2: Percentage of fully protected against symptomatic HAV infection with (A) 1-dose and (B) 2-doses. Red color do not consider additional protection against symptomatic infection and infectiousness after waning of protection; Black color consider 10 years additional mean duration of partial protection due to Immune Memory Protection; blue color 20 years; green color 50 years and cyan color 100 years.**


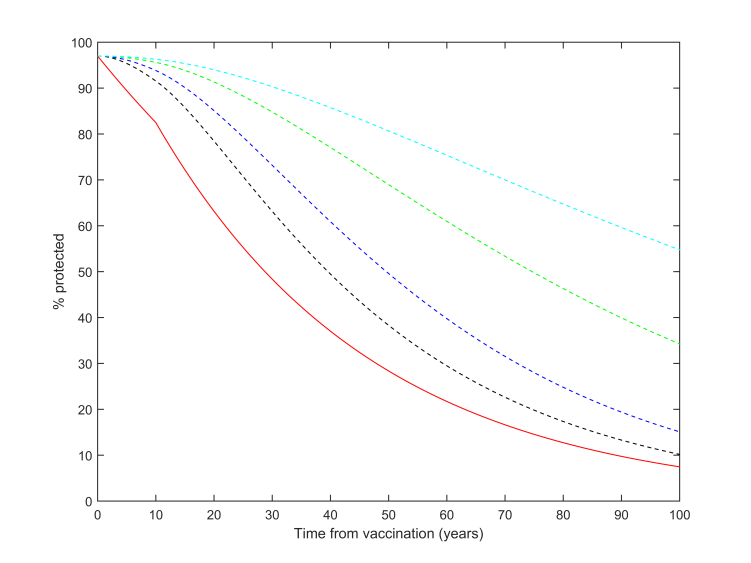


A


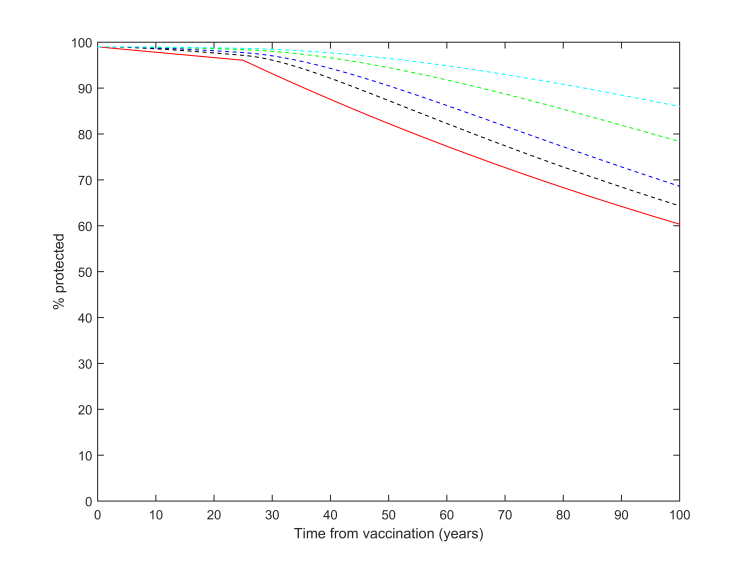


B

%: percentage; HAV: Hepatitis A Virus

***Differences between Carlos et al. ^3^ and SAS model results for the Non-vaccinated Group***

The epidemiological model (i.e. hepatitis A and icteric infections) was developed in *Matlab* (7.9.0 software). The original health economic model was developed in *TreeAge Pro 2013*, whereas the current analysis was developed in *SAS* (Version 9.2). Some differences existed in the calculations primarily regarding the discounted treatment costs (Supplementary Table 1).

**Supplementary Table 1. Comparison of outcomes between Carlos et al. and *SAS* model results for the base case, in the non-vaccinated group.**

| **Projected HAV cases/costs** | ***Carlos et al. ^3^*** | ***SAS* Model** | **difference** | **% difference** |
| --- | --- | --- | --- | --- |
| **Total HAV infections** | 46,841,960 | 46,842,027 | 67 | <0.01% |
| **Anicteric infections** | 31,664,073 | 31,664,135 | 62 | <0.01% |
| Asymptomatic | 15,832,057 | 15,832,067 | 10 | <0.01% |
| Symptomatic | 15,832,057 | 15,832,067.5 | 10.5 | <0.01% |
| **Icteric infections** | 15,177,887 | 15,177,892 | 5 | 0.00% |
| Reported | 1,022,924 | 1,022,924 | 0 | 0.00% |
| Hospitalization | 53,153 | 53,153 | 0 | 0.00% |
| Fulminant hepatitis | 6,103 | 6,103 | 0 | 0.00% |
| Alive after liver transplant | 275 | 275 | 0 | 0.00% |
| Death | 3,710 | 3,709 | -1 | -0.03% |
| **Costs and QALYs, (Mexican pesos)** |  |  |  |  |
| Discounted Medical treatment costs | 12,556,191,424 | 12,573,978,804 | 17,787,380 | <0.01% |
| QALYs Lost | 374,689 | 376,386 | 1,697 | 0.45% |

**HAV, Hepatitis A Virus; QALY, quality-adjusted life-year**

A previously published MSLIR (maternal antibody protection, susceptible, latent, infectious, recovered) dynamic model of hepatitis A in Mexico was adapted to account for the so called immune memory protection (IMP).**^4^** In the original model, HAV vaccinated individuals were assumed to have an all or none efficacy against HAV infection only with a pre-defined waning of vaccine protection over time. In the sequel, the original model was adapted by adding a new IMP state wherein the vaccinated individuals were partially protected by IMP after the vaccine efficacy against infection had waned. In the IMP state individuals were assumed to have no protection against infection anymore but were assumed to have a lower probability of developing an icteric infection and a potential a reduction in infectiousness as well. IMP was assumed to wane after a mean duration of 10, 20 or 50 years, after which the individuals have the same status as none vaccinated individuals.

**Supplementary Figure 3: Adapted Epidemiological Model Structure.**

**
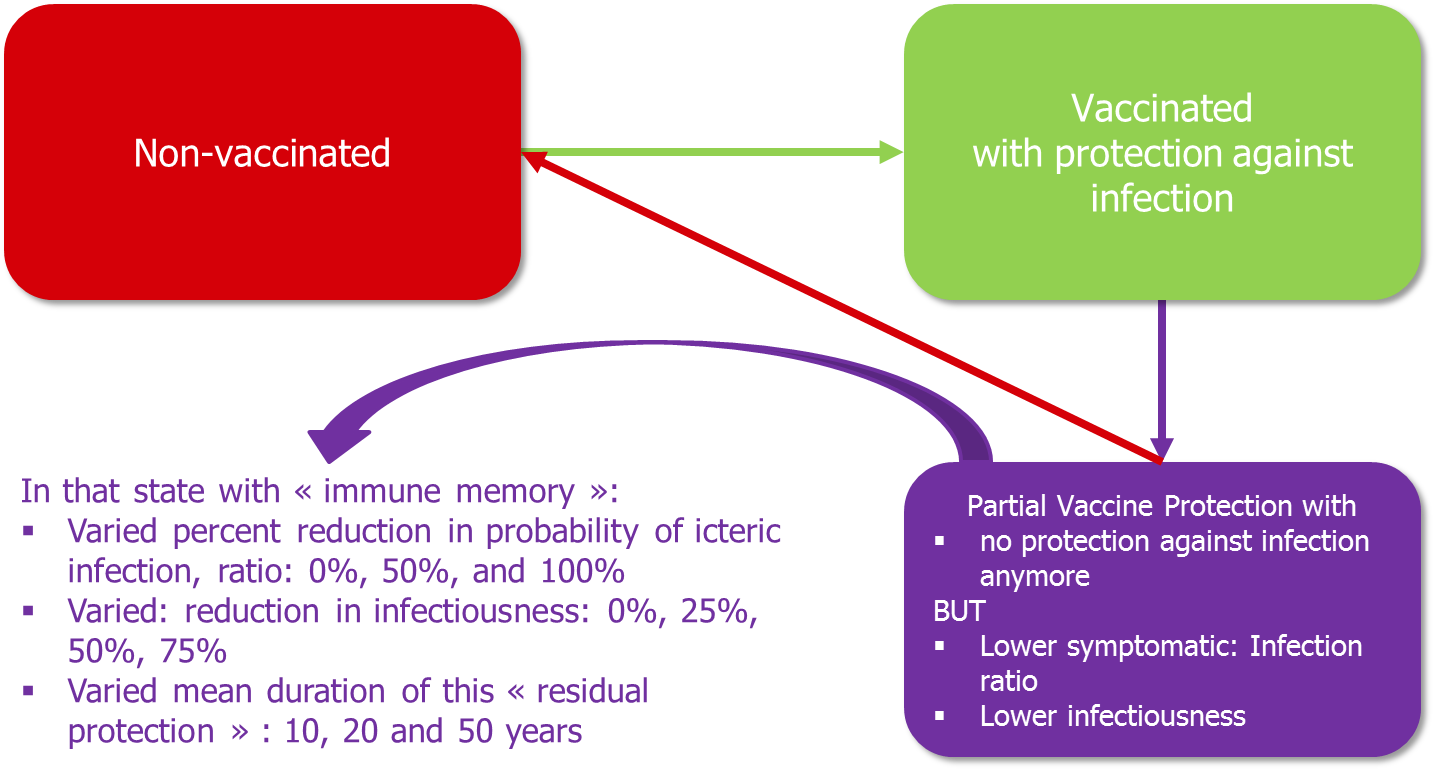
**

**References**

1. Armstrong GL, Bell BP. Hepatitis A virus infections in the United States: model-based estimates and implications for childhood immunization. Pediatrics. 2002; 109:839-845.
2. Ellis A, Ruttimann RW, Jacobs RJ Meyerhoff AS, Innis BL. Cost-effectiveness of childhood hepatitis A vaccination in Argentina: a second dose is warranted. Rev Panam Salud Publica. 2007; 21:345-56
3. Carlos F, Gomez JA, Anaya P, Romano-Mazzotti L. Health economic assessment of universal immunization of toddlers against Hepatitis A virus (HAV) in Mexico. Hum Vaccin Immunother 2015; 26:0. [Epub ahead of print].
4. Van Effelterre T, De Antonio-Suarez R, Cassidy A Romano-Mazotti L, Marano C. Model-based projections of the population-level impact of hepatitis A vaccination in Mexico. Hum Vaccin Immunother 2012; 8:1099-1108.
